# Does Acceptance Aid Mental Health? A National Survey Following the 2023 Turkey‐Syria Earthquake

**DOI:** 10.1002/smi.70133

**Published:** 2025-12-28

**Authors:** Tiffany Junchen Tao, Crystal Jingru Li, Huinan Liu, Aysuhan Tuba Saral, Robin Goodwin, Wai Kai Hou

**Affiliations:** ^1^ Centre for Psychosocial Health The Education University of Hong Kong Hong Kong SAR China; ^2^ Department of Psychology University of California Irvine California USA; ^3^ Department of Psychology The Education University of Hong Kong Hong Kong SAR China; ^4^ Department of Special Education and Counselling The Education University of Hong Kong Hong Kong SAR China; ^5^ Department of International Education The Education University of Hong Kong Hong Kong SAR China; ^6^ Department of Psychology University of Warwick Coventry England

**Keywords:** acceptance, disaster, integration, psychiatric symptoms

## Abstract

Natural disasters threaten population mental health. Acceptance, denoting recognition and assent of the finality of a situation, is a coping strategy that has been recommended following major disasters. This study investigates this topic for which existing findings are mixed. A survey was administered among a population‐representative Turkish national sample (*N* = 7585) recruited through the internet panel of TGM Research (September–October 2023). We examined how acceptance and its interaction with cognitive integration (i.e., finding sense in a stressful event and finding significance in life after the event) were associated with common psychiatric symptoms. Respondents with high acceptance showed *higher* symptoms of anxiety, depression and PTSD. These associations were stronger when respondents also reported low perceived significance in life after the event. In the post‐disaster phase, the adaptiveness of acceptance could interplay with other factors, such as how much an individual finds significance in life following the disaster.

## 1 Introduction

Natural disasters threaten population mental health. The 7.8‐magnitude earthquake along the Turkey‐Syria border in 2023 has damaged lives and properties and left a severe mental health burden. Around 50% of the respondents exhibited probable anxiety, depression, or PTSD 8 months after the earthquake, with higher sociopolitical vulnerability factors exacerbating the psychiatric consequences of earthquake exposure (Hou et al. 2024).

Post‐disaster medical and public health services have called for leveraging any existing resources to promptly address the pressing mental health needs among affected populations. Lay providers were suggested to receive training to deliver psychological first aid support, and survivors were suggested to learn self‐help coping techniques. Acceptance denotes recognition and assent of the finality of a situation, and is a commonly recommended coping strategy across regions and sociocultural contexts following major disasters (Williams et al. 2009). There is evidence suggesting that acceptance in the face of stressful life events could promote passive and stoic responses (Maddi and Hightower 1999). Among 1270 trauma‐exposed undergraduate students, frequent adoption of acceptance was associated with higher PTSD symptom severity (Kearns et al. 2021). In a study of 115 older US adults during COVID‐19, the inverse association between perceived stress and physical health was significant only at higher but not lower levels of acceptance (Tracy et al. 2022), meaning that lower acceptance potentially buffered against the physical health effects of stress. There was also a prior study revealing the multifaceted feature of a measure for acceptance (Lyne and Roger 2000).

Some researchers suggest that acceptance in its adaptive form may accompany successful meaning‐making efforts (Quinto et al. 2022). Traumatic events could introduce new information that is inconsistent with an individual's pre‐trauma worldview system and basic assumptions. Cognitive integration is the meaning‐making process to reconcile the discrepancies between trauma‐related information and prior life experience, and in some ways indicates the person has successfully digested this experience (Horowitz 1986; Joseph et al. 2012; Joseph and Linley 2005; Park 2010). Integration more specifically includes comprehensibility (i.e., finding sense in the stressful event) and footing in the world (i.e., finding significance in life following the event) (George and Park 2016; Martela and Steger 2016). Together, the process of integration results in an updated worldview system that is in line with the individual's actual experience. As an example, in a study of expressive writing (Margola et al. 2010), bereaved adolescents who expressed more signs of processing and making sense of the event showed stably low posttraumatic stress symptoms or recovery of the symptoms from clinical to nonclinical levels over a 4‐month period. In another systematic review on coping with chronic diseases, acceptance of cancer was positively correlated with cognitive integration (Quinto et al. 2022).

To our knowledge, limited studies to date have explicitly investigated whether acceptance as a coping strategy is adaptive or maladaptive within the context of large‐scale disasters. The current study therefore aims to investigate (a) the associations between acceptance coping and common psychiatric symptoms and (b) the potential interaction effects of cognitive integration in a nationally representative Turkish sample following the 2023 Turkey‐Syria Earthquake.

## 2 Methods

### 2.1 Respondents and Procedures

Our sample (*N* = 7585) was recruited through the internet panel of TGM Research (September–October 2023), following the Ethics Committee's approval from The Education University of Hong Kong. Respondents were eligible if they were a Turkish resident aged 18 years or above who were Turkish‐speaking. Written consent was provided. Attention checks were implemented to ensure data accuracy.

### 2.2 Measures

To measure *acceptance*, respondents were shown the instructions, ‘The following items describe how people respond when they confront difficult or stressful events in their lives. Please indicate your responses when you experience stress *due to the 2023 Turkey‐Syria* *Earthquake*.’ They completed the 4‐item Acceptance subscale on the Turkish version of the COPE Inventory (e.g., ‘I get used to the idea that this happened.’), on a 4‐point scale (1 = I usually don't do this at all, 4 = I usually do this a lot) (Baki 2018) (*α* = 0.830). While the acceptance scale can be adapted to capture either a trait or state, the scale in our study captured state acceptance that is more specific to how people cope with the earthquake. A total score was calculated (range: 4–16), with higher scores indicating higher acceptance.

*Cognitive integration*, including the *comprehensibility* (e.g., ‘I have difficulty integrating this event into my understanding about the world.’) (*α* = 0.773) and *footing in the world* (e.g., ‘This event has made me feel less purposeful.’) (*α* = 0.844) subscales, was measured with the 6‐item Integration of Stressful Life Experiences Scale–Short Form, on a 4‐point Likert scale (1 = Strongly disagree, 4 = Strongly agree) (Holland et al. 2014). Two sub‐scores were calculated following reverse coding (ranges: 3–12), with higher scores indicating higher levels of comprehensibility and footing in the world.

*Symptoms* of anxiety, depressive, and earthquake‐induced PTSD were assessed using the 7‐item Generalized Anxiety Disorder scale (Konkan et al. 2013) (*α* = 0.921), the 9‐item Patient Health Questionnaire (Levis et al. 2019) (*α* = 0.899), and the 6‐item PTSD Checklist–Specific Version (Kocabaşoğlu et al. 2005; Lang et al. 2012) (*α* = 0.889), respectively. Total scores were calculated (anxiety range: 0–21; depression range: 0–27; PTSD range: 6–30), with higher scores indicating higher symptoms.

*Demographic information* was collected with a standardized proforma, assessing age, gender, marital status, and socioeconomic status (education level, employment status, and monthly household income). *Disaster exposure* was indicated by proximity to affected area (in non‐affected vs. affected area), property damage (no vs. yes), and interpersonal loss (no vs. yes).

### 2.3 Analytic Plan

All missing data (< 1%) were handled with multiple imputation. Preliminary analyses were conducted to explore the associations between acceptance, cognitive integration (comprehensibility, footing in the world), and symptoms. For main analyses, moderation models were constructed in M*plus*, with acceptance as the focal predictor, two aspects of cognitive integration (comprehensibility, footing in the world) as the moderators, and symptoms as the outcomes, controlling for demographic covariates (age, gender, marital status). Predictor, moderators, and outcomes were standardized. Additional subgroup analyses included stratifications by socioeconomic status and disaster exposure (proximity to affected area, property damage, or interpersonal loss). Difference tests were run to compare path coefficients across subgroups. Population weight was applied based on age, gender, and geographical region.

## 3 Results

Respondents were mostly aged 18–29 (41.82%) or 30–44 years (38.44%), males (52.37%), married (53.57%), employed (73.55%), had a tertiary education or above (69.69%), and had high or middle monthly household income (81.71%). They also showed a relatively high level of psychiatric symptoms (anxiety: *M* = 7.73, SD = 5.24, range = 0–21; depression: *M* = 9.60, SD = 6.09, range = 0–27; PTSD: *M* = 13.93, SD = 5.69, range = 6–30), with altogether 4453 (58.71%) showing probable anxiety, depression, and/or PTSD (based on validated clinical cutoffs). Symptoms were higher among individuals with higher disaster exposure, namely in affected regions (19.62% of the entire sample), with property damage (4.89%) and with interpersonal loss (21.02%) (Supporting Information S1: Material 1). Our sample therefore included a relatively high proportion of affected individuals with probable psychiatric conditions and thus a need for psychosocial support. Table 1 provides a full summary of sample descriptives. Bivariate correlations showed that acceptance was positively whereas cognitive integration negatively associated with all symptoms (Supporting Information S1: Material 2).

**TABLE 1:** Descriptive characteristics of the current sample (*N* = 7585).

Variable	*n* (%)
Age	
18–29	3172 (41.82)
30–44	2916 (38.44)
45 or above	1497 (19.74)
Gender	
Female	3613 (47.63)
Male	3972 (52.37)
Marital status	
Single, divorced or widowed	3522 (46.43)
Married	4063 (53.57)
Education level	
Secondary or below	2299 (30.31)
Tertiary or above	5286 (69.69)
Employment	
Unemployed or dependent	2006 (26.45)
Employed	5579 (73.55)
Monthly household income^a^	
Low income	1387 (18.29)
High or middle income	6198 (81.71)
Proximity to affected area^b^	
In affected area	1488 (19.62)
In non‐affected area	6097 (80.38)
Housing damage status	
Yes	371 (4.89)
No	7214 (95.11)
Interpersonal loss status^c^	
Yes	1594 (21.02)
No	5991 (78.98)
Acceptance, *M* (SD) [range: 4–16]	10.65 (3.14)
Cognitive integration—Comprehensibility, *M* (SD) [range: 3–12]	7.81 (2.20)
Cognitive integration—Footing in the world, *M* (SD) [range: 3–12]	8.21 (2.41)
Psychiatric symptoms	
Anxiety symptoms, *M* (SD) [range: 0–21]	7.73 (5.24)
Depressive symptoms, *M* (SD) [range: 0–27]	9.60 (6.09)
PTSD symptoms, *M* (SD) [range: 6–30]	13.93 (5.69)
Probable psychiatric conditions^d^	
Probable anxiety	2353 (31.02)
Probable depression	3349 (44.15)
Probable PTSD	3619 (47.71)
Probable anxiety, depression, or PTSD	4453 (58.71)

^a^ Low monthly household income was defined as 11,499 TL (approximately 382 USD) or below, with reference to the net minimum wage in effect in July 2023.

^b^ Affected area included Adana, Adiyaman, Diyarbakir, Elaziğ, Gaziantep, Hatay, Kilis, Kahramanmaraş, Malatya, Osmaniye, and Şanliurfa.

^c^ Loss targets included family, relatives, friends, or someone the respondents know.

^d^ Probable anxiety was defined as a score of 10 or above on the 7‐item Generalized Anxiety Disorder scale (GAD‐7); probable depression was defined as a score of 10 or above on the 9‐item Patient Health Questionnaire (PHQ‐9); probable PTSD was defined as a score of 14 or above on the 6‐item PTSD Checklist—Specific Version (PCL‐6‐S).

The moderation model displayed good data‐model fit (RMSEA = 0.048, 90% CI [0.043, 0.053], SRMR = 0.032, CFI = 0.977, TLI = 0.962) (Table 2). Acceptance was positively associated with symptoms of anxiety (*β* = 0.072, 95% CI [0.050, 0.094]), depression (*β* = 0.093, 95% CI [0.069, 0.117]) and PTSD (*β* = 0.093, 95% CI [0.069, 0.116]), while comprehensibility and footing in the world were both inversely associated with symptoms of anxiety (*β* = −0.207, 95% CI [–0.233, −0.182]; *β* = −0.419, 95% CI [–0.444, −0.394]), depression (*β* = −0.154, 95% CI [–0.182, −0.127]; *β* = −0.364, 95% CI [–0.391, −0.338]) and PTSD (*β* = −0.194, 95% CI [–0.221, −0.167]; *β* = −0.400, 95% CI [–0.426, −0.374]).

**TABLE 2:** Associations between acceptance, cognitive integration, and psychological symptoms.

Dependent variables	Predictors	Estimate *β* [95% CI]
Anxiety symptoms (*Y*_1_)	Acceptance (*X*)	0.072 [0.050, 0.094]***
	Comprehensibility (*W*_1_)	−0.207 [–0.233, −0.182]***
	Footing in the world (*W*_2_)	−0.419 [–0.444, −0.394]***
	Acceptance × Comprehensibility (*XW*_1_)	0.009 [–0.023, 0.042]
	**Acceptance × Footing in the world (*XW***_**2**_ **)**	**−0.076 [–0.110, –0.043]** ***
Depressive symptoms (*Y*_2_)	Acceptance (*X*)	0.093 [0.069, 0.117]***
	Comprehensibility (*W*_1_)	−0.154 [–0.182, −0.127]***
	Footing in the world (*W*_2_)	−0.364 [–0.391, −0.338]***
	**Acceptance × Comprehensibility (*XW***_**1**_ **)**	**0.053 [0.019, 0.087]** **
	**Acceptance × Footing in the world (*XW***_**2**_ **)**	**−0.096 [–0.131, –0.061]** ***
PTSD symptoms (*Y*_3_)	Acceptance (*X*)	0.093 [0.069, 0.116]***
	Comprehensibility (*W*_1_)	−0.194 [–0.221, −0.167]***
	Footing in the world (*W*_2_)	−0.400 [–0.426, −0.374]***
	Acceptance × Comprehensibility (*XW*_1_)	0.008 [–0.026, 0.041]
	**Acceptance × Footing in the world (*XW***_**2**_ **)**	**−0.082 [–0.118, –0.046]** ***

*Note:* Bolded texts signal the presence of an interaction effect. Analyses were based on the MLR estimator. Analyses were weighted. Model fit indices: RMSEA = 0.048, 90% CI [0.043, 0.053], SRMR = 0.032, CFI = 0.977, TLI = 0.962, χ^2^(16) = 294.368. Acceptance and cognitive integration (comprehensibility, footing in the world), and symptoms were included as continuous variables and were standardised. Demographic covariates included age, gender, and marital status.

^**^ *p* < 0.010.

^***^ *p* < 0.001.

The acceptance × footing in the world interaction was significant for all symptoms (*β*s ranged from −0.096 [–0.131, −0.061] to −0.076 [–0.110, −0.043]), whereas the acceptance × comprehensibility interaction was significant for depressive symptoms (*β* = 0.053 [0.019, 0.087]). Simple slope analyses (Table 3) revealed that the positive acceptance‐symptom associations were *strongest* when footing in the world was *low* (anxiety: *β* = 0.137, 95% CI [0.096, 0.177]; depressive: *β* = 0.174, 95% CI [0.131, 0.218]; PTSD: *β* = 0.162, 95% CI [0.118, 0.205]), comparatively *weaker* when footing in the world was *medium* (anxiety: *β* = 0.071, 95% CI [0.049, 0.093]; depressive: *β* = 0.092, 95% CI [0.068, 0.116]; PTSD: *β* = 0.092, 95% CI [0.069, 0.115]), and *non‐significant* when footing in the world was *low*. In contrast, the acceptance‐depressive symptoms association was *stronger* as the level of comprehensibility *increased* (low: *β* = 0.049, 95% CI [0.010, 0.088]; medium: *β* = 0.092, 95% CI [0.068, 0.116]; high: *β* = 0.136, 95% CI [0.101, 0.170]).

**TABLE 3:** Simple slopes for acceptance and psychological symptoms, at different levels of cognitive integration.

	Estimate *β* [95% CI] Cognitive integration					
	Comprehensibility			Footing in the world		
	Low	Medium	High	Low	Medium	High
Acceptance → Anxiety symptoms	—	—	—	0.137 [0.096, 0.177]	0.071 [0.049, 0.093]	n.s.
Acceptance → Depressive symptoms	0.049 [0.010, 0.088]	0.092 [0.068, 0.116]	0.136 [0.101, 0.170]	0.174 [0.131, 0.218]	0.092 [0.068, 0.116]	n.s.
Acceptance → PTSD symptoms	—	—	—	0.162 [0.118, 0.205]	0.092 [0.069, 0.115]	n.s.

*Note:* All presented simple slopes were significant, unless otherwise specified. ‘—’ Indicates an absence of significant interaction to probe simple slopes.

Abbreviation: n.s. = not statistically significant.

Stratified moderation (Supporting Information S1: Materials 3–6) revealed how the results could differ between subgroups. Among individuals with high SES, acceptance and footing in the world were more strongly associated with lower depressive symptoms, whereas among individuals with low SES, the acceptance‐depressive symptoms association was attenuated to a greater extent with increasing footing in the world. In addition, footing in the world or comprehensibility were more strongly associated with lower PTSD symptoms for individuals with high SES, in the affected area, or with interpersonal loss.

## 4 Discussion

In our study of 7585 Turkish participants after the Turkey‐Syria Earthquake, acceptance was positively, whereas both aspects of cognitive integration (comprehensibility, footing in the world) were inversely, associated with anxiety, depressive, and PTSD symptoms. Additionally, the acceptance‐symptoms associations *decreased* as footing in the world increased, whereas the acceptance‐depressive symptoms association *increased* as comprehensibility increased.

Our study adds new findings to the discussion around the adaptive versus maladaptive nature of acceptance as a coping strategy, particularly within the relatively earlier phase following large‐scale disasters such as the current earthquake. Consistent with some other work, acceptance was observed to either positively relate to psychiatric symptoms (Garnefski and Kraaij 2006; Kearns et al. 2021) or amplify the physical health consequences of stress (Tracy et al. 2022). The disparate evidence on the mental health benefit of acceptance could be due in part to different types of acceptance, namely active versus passive/resigned acceptance: The former refers to recognizing the negative situation and handling challenges in a constructive manner, whereas the latter abandoning actions coupled with negative future expectations (Nakamura and Orth 2005). Active acceptance has been associated with positive personalities/outcomes such as optimism, hardiness, and posttraumatic growth, whereas passive/resigned acceptance with negative ones such as denial coping and psychiatric symptoms (Maddi and Hightower 1999).

In our study, the COPE Acceptance subscale captured the passive/resigned type of acceptance. There could be two explanations for the current findings: either being in the state of acceptance was fundamentally maladaptive in the current context, or respondents assigned a negative connotation to the idea of acceptance. Coping strategies dynamically function across temporal and situational contexts (Bonanno and Burton 2013; Scheier et al. 1986; Shing et al. 2016; Troy et al. 2013). For example, relative to acceptance, distraction might sometimes be preferred within highly stressful contexts in the short‐term, and problem‐solving might be preferred when it is possible for individuals to regain a sense of agency through managing a controllable event. In the current earthquake, amongst other crises, there could be things people are willing and capable to do, such as keeping up with disaster information and tips, leading a healthy lifestyle, as well as directly or indirectly helping affected others, whereas overreliance on acceptance over potentially constructive alternatives might lead to learnt helplessness and psychological distress. It was also possible that items tapping into acceptance (e.g., ‘I accept that this has happened and that it can't be changed.’) could have been understood as a fatalistic response in some cultures (Nageeb et al. 2018; Yeh et al. 2006), and fatalism was in turn associated with more negative health behaviours and outcomes (Franklin et al. 2007). Acceptance as an individualistic coping strategy may also be less preferred in collectivistic cultures (Fischer et al. 2010).

In the meantime, our study illustrates the further implication of cognitive integration in understanding the acceptance‐symptoms associations. Notably, the significant acceptance‐footing in the world interaction suggested that for individuals who struggled to find significance in life following the disaster, resigned acceptance was particularly strongly associated with symptoms; in contrast, for those who perceived a strong significance in life, acceptance was no longer associated with symptoms. Indeed, why we were observing this pattern may have multiple explanations. It is possible that reorienting oneself to a life post‐disaster may buffer against resignation, in line with a well‐established understanding of the positive outcomes of significance finding (George and Park 2016; Martela and Steger 2016; Park 2010). This phenomenon is also present in the literature. In one study involving individuals with cardiac disease receiving ICD implantations, patients who were able to incorporate the device into their newly created self‐identities coped better than those who passively approached this fact (Humphreys et al. 2016). Relatedly, in another qualitative interview on 18 patients with end‐stage renal failure, those who actively accepted reality got back to life and moved on through making adaptive accommodations for their illness and treatment (Wright and Kirby 1999).

Interestingly, the acceptance‐comprehensibility interaction instead showed that when an individual reported making more sense of the disaster, the acceptance‐depressive symptoms link was more pronounced. While comprehensibility is generally desirable, one possible explanation for this seemingly counterintuitive observation is that individuals do not always assimilate the event in a balanced way, and may sometimes biasedly internalize blame or distort the meaning of the event to fit preexisting beliefs, leading to maladaptive outcomes (Held et al. 2025). However, as this pattern was not also seen for anxiety nor PTSD symptoms, the interpretation may be preliminary and should be treated with caution.

Finally, our supplementary analyses point to the value of considering the contextual role of socioeconomic status and disaster exposure. This is in itself an important issue, as existing work has suggested that social context and disaster‐related characteristics could shape not only people's tendency to adopt certain coping strategies over others, but they could also determine the relative utility of these strategies given their fit with the stressful situation (Chen and Miller 2012; Shing et al. 2016). This further echoes an earlier point that the functions of coping strategies could vary.

Our results provided population‐representative evidence on the adaptive utility of coping through acceptance. However, a few limitations should be noted. The design was cross‐sectional, causal inferences were not supported, symptoms were self‐reported, and acceptance scales other than the COPE were not used. Notwithstanding these limitations, the key takeaway from our results is the importance to consider acceptance not as a uniformly adaptive or maladaptive construct, but to consider its adaptive utility as potentially context‐dependent. To further clarify the nature of function of different types of acceptance under different contexts, future research could simultaneously investigate different acceptance scales, different coping scales (other than acceptance) and their associations with psychological adaptation following different disasters (which ideally vary across geographical, temporal, and cultural contexts). A few other outstanding questions concern why comprehensibility and footing in the world interact with acceptance differently, and why the associations between acceptance, integration, and symptoms may differ by subgroups (e.g., socioeconomic status, disaster exposure level). Answers to these questions will be of interest to researchers and practitioners alike.

## Author Contributions

**Tiffany Junchen Tao:** conceptualization, data curation, formal analysis, investigation, methodology, writing – original draft, writing – review and editing. **Crystal Jingru Li:** data curation, writing – review and editing. **Huinan Liu:** data curation, writing – review and editing. **Aysuhan Tuba Saral:** conceptualization, investigation, writing – review and editing. **Robin Goodwin:** conceptualization, writing – review and editing. **Wai Kai Hou:** conceptualization, data curation, formal analysis, funding acquisition, investigation, methodology, project administration, resources, supervision, writing – original draft, writing – review and editing.

## Funding

This work was supported by Research Grants Council, University Grants Committee, Hong Kong SAR, China [Grant No. 18600320 (W.K.H.)].

## Ethics Statement

This study received ethics approval from the Human Research Ethics Committee at The Education University of Hong Kong (Reference No.: 2022‐2023‐0435).

## Consent

Written consent was obtained from all respondents prior to their study participation.

## Conflicts of Interest

The authors declare no conflicts of interest.

## Supporting information


Supporting Information S1

## Data Availability

The data that support the findings of this study are available from the corresponding author upon reasonable request.
