# Supplementary material for: Does Acceptance Aid Mental Health? A National Survey Following the 2023 Turkey‐Syria Earthquake
Source: Stress Health. 2025 Dec 28;42(1):e70133. doi: 10.1002/smi.70133 (PMC12765484; doi:10.1002/smi.70133)
Supplement: Supplementary file 1 — Supporting Information S1 [file SMI-42-e70133-s001.docx]

**Supplementary Material 1**

*Psychological Symptoms by Degree of Disaster Impact*

|  | **Anxiety symptoms** | | **Depressive symptoms** | | **PTSD symptoms** | |
| --- | --- | --- | --- | --- | --- | --- |
|  | *M* (*SD*) | *p* | *M* (*SD*) | *p* | *M* (*SD*) | *p* |
| **Proximity to affected area** |  | <.001 |  | <.001 |  | <.001 |
| In non-affected area (*n*=6,097) | 7.48 (5.21) |  | 9.26 (6.02) |  | 13.46 (5.54) |  |
| In affected area (*n*=1,488) | 8.75 (5.25) |  | 10.98 (6.19) |  | 15.85 (5.92) |  |
| **Property damage status** |  | <.001 |  | <.001 |  | <.001 |
| No damage (*n*=7,214) | 7.59 (5.20) |  | 9.45 (6.04) |  | 13.74 (5.61) |  |
| Damage (*n*=371) | 10.31 (5.33) |  | 12.60 (6.28) |  | 17.58 (6.03) |  |
| **Interpersonal loss status** |  | <.001 |  | <.001 |  | <.001 |
| No loss (*n*=5,991) | 7.19 (5.08) |  | 8.91 (5.87) |  | 13.15 (5.35) |  |
| Loss (*n*=1,594) | 9.74 (5.36) |  | 12.19 (6.21) |  | 16.86 (5.97) |  |

**Supplementary Material 2**

*Bivariate Correlations Between Test Variables*

|  | 1 | 2 | 3 | 4 | 5 |
| --- | --- | --- | --- | --- | --- |
| 1. Acceptance | – |  |  |  |  |
| 2. Cognitive integration – Comprehensibility | –.157 | – |  |  |  |
| 3. Cognitive Integration – Footing in the world | –.063 | .657 | – |  |  |
| 4. Anxiety symptoms | .115 | –.499 | –.572 | – |  |
| 5. Depressive symptoms | .126 | –.412 | –.486 | .762 | – |
| 6. PTSD symptoms | .128 | –.479 | –.553 | .726 | .713 |

*Notes*. All correlations were significant at *p*<.001 level.

**Supplementary Material 3**

*Associations Between Acceptance, Cognitive Integration, and Psychological Symptoms (Stratified by Socioeconomic Status)*

| **Dependent variables** | **Predictors** | **Socioeconomic status** | | |
| --- | --- | --- | --- | --- |
|  |  | **High (*n*=3,779)**  ***β*_1_ [95% CI]** | **Low (*n*=3,806)**  ***β*_2_ [95% CI]** | **Difference test**  ***b*_1_ *– b*_2_ [95% CI]** |
| Anxiety symptoms (*Y*_1_) | Acceptance (*X*) | 0.054 [0.023, 0.085]** | 0.091 [0.061, 0.123]*** | –0.037 [–0.080, 0.007] |
|  | Comprehensibility (*W*_1_) | –0.205 [–0.241, –0.170]*** | –0.209 [–0.244, –0.172]*** | 0.002 [–0.049, 0.053] |
|  | Footing in the world (*W*_2_) | –0.435 [–0.471, –0.400]*** | –0.406 [–0.444, –0.371]*** | –0.031 [–0.083, 0.020] |
|  | Acceptance *×* Comprehensibility (*XW*_1_) | 0.001 [–0.045, 0.048] | 0.018 [–0.027, 0.063] | –0.013 [–0.066, 0.039] |
|  | Acceptance *×* Footing in the world (*XW*_2_) | –0.050 [–0.099, –0.002]* | –0.099 [–0.144, –0.053]*** | 0.040 [–0.017, 0.097] |
| Depressive symptoms (*Y*_2_) | **Acceptance (*X*)** | **0.068 [0.034, 0.102]***** | **0.121 [0.088, 0.155]***** | **–0.052 [–0.100, –0.004]*** |
|  | Comprehensibility (*W*_1_) | –0.162 [–0.201, –0.124]*** | –0.147 [–0.183, –0.109]*** | –0.017 [–0.071, 0.038] |
|  | **Footing in the world (*W*_2_)** | **–0.394 [–0.432, –0.356]***** | **–0.340 [–0.381, –0.305]***** | **–0.056 [–0.109, –0.002]*** |
|  | Acceptance *×* Comprehensibility (*XW*_1_) | 0.047 [–0.002, 0.096] | 0.058 [0.012, 0.105]* | –0.009 [–0.064, 0.046] |
|  | **Acceptance *×* Footing in the world (*XW*_2_)** | **–0.052 [–0.103, –0.000]*** | **–0.134 [–0.180, –0.087]***** | **0.068 [0.009, 0.128]*** |
| PTSD symptoms (*Y*_3_) | Acceptance (*X*) | 0.086 [0.054, 0.118]*** | 0.100 [0.067, 0.135]*** | –0.014 [–0.060, 0.032] |
|  | Comprehensibility (*W*_1_) | –0.181 [–0.219, –0.144]*** | –0.206 [–0.243, –0.167]*** | 0.023 [–0.031, 0.077] |
|  | **Footing in the world (*W*_2_)** | **–0.433 [–0.469, –0.398]***** | **–0.369 [–0.410, –0.330]***** | **–0.066 [–0.120, –0.012]*** |
|  | Acceptance *×* Comprehensibility (*XW*_1_) | 0.004 [–0.042, 0.049] | 0.009 [–0.040, 0.058] | –0.005 [–0.059, 0.050] |
|  | Acceptance *×* Footing in the world (*XW*_2_) | –0.054 [–0.103, –0.005]* | –0.103 [–0.154, –0.052]*** | 0.041 [–0.020, 0.101] |

* *p*<.050, ** *p*<.010, *** *p*<.001.

*Notes*. Bolded texts signal the presence of a between-group difference in the corresponding path coefficient. *β* is standardized path coefficient; *b* is unstandardized path coefficient. Analyses were based on the MLR estimator. Analyses were weighted. Model fit indices: RMSEA=0.049, 90% CI [0.044, 0.054], SRMR=0.033, CFI=0.977, TLI=0.961, χ²(32)=319.739. Acceptance, cognitive integration (comprehensibility, footing in the world), and symptoms were included as continuous variables and were standardized. Socioeconomic status was categorized as high (i.e., high education, employment, and high income) and low (i.e., low education, non-employment, or low income). Demographic covariates included age, gender, and marital status.

**Supplementary Material 4**

*Associations Between Acceptance, Cognitive Integration, and Psychological Symptoms (Stratified by Proximity to Affected Area)*

| **Dependent variables** | **Predictors** | **Proximity to affected area** | | |
| --- | --- | --- | --- | --- |
|  |  | **Non-affected area (*n*=6,097)**  ***β*_1_ [95% CI]** | **Affected area (*n*=1,488)**  ***β*_2_ [95% CI]** | **Difference test**  ***b*_1_ *– b*_2_ [95% CI]** |
| Anxiety symptoms (*Y*_1_) | Acceptance (*X*) | 0.078 [0.053, 0.103]*** | 0.045 [–0.004, 0.093] | 0.033 [–0.021, 0.087] |
|  | Comprehensibility (*W*_1_) | –0.208 [–0.236, –0.180]*** | –0.209 [–0.269, –0.148]*** | 0.001 [–0.066, 0.069] |
|  | Footing in the world (*W*_2_) | –0.405 [–0.433, –0.376]*** | –0.445 [–0.502, –0.389]*** | 0.042 [–0.022, 0.107] |
|  | Acceptance *×* Comprehensibility (*XW*_1_) | 0.010 [–0.027, 0.046] | 0.016 [–0.057, 0.090] | –0.005 [–0.071, 0.061] |
|  | Acceptance *×* Footing in the world (*XW*_2_) | –0.087 [–0.126, –0.049]*** | –0.050 [–0.120, 0.020] | –0.034 [–0.100, 0.032] |
| Depressive symptoms (*Y*_2_) | Acceptance (*X*) | 0.095 [0.068, 0.122]*** | 0.079 [0.025, 0.133]** | 0.016 [–0.044, 0.075] |
|  | Comprehensibility (*W*_1_) | –0.146 [–0.176, –0.115]*** | –0.198 [–0.261, –0.135]*** | 0.052 [–0.018, 0.123] |
|  | Footing in the world (*W*_2_) | –0.354 [–0.384, –0.324]*** | –0.358 [–0.416, –0.300]*** | 0.005 [–0.061, 0.072] |
|  | Acceptance *×* Comprehensibility (*XW*_1_) | 0.058 [0.020, 0.095]** | 0.043 [–0.037, 0.124] | 0.013 [–0.058, 0.084] |
|  | Acceptance *×* Footing in the world (*XW*_2_) | –0.107 [–0.146, –0.067]*** | –0.060 [–0.137, 0.017] | –0.042 [–0.114, 0.029] |
| PTSD symptoms (*Y*_3_) | Acceptance (*X*) | 0.097 [0.071, 0.124]*** | 0.070 [0.019, 0.122]** | 0.027 [–0.030, 0.084] |
|  | **Comprehensibility (*W*_1_)** | **–0.182 [–0.211, –0.152]***** | **–0.266 [–0.330, –0.201]***** | **0.084 [0.013, 0.155]*** |
|  | Footing in the world (*W*_2_) | –0.398 [–0.427, –0.369]*** | –0.337 [–0.399, –0.274]*** | –0.060 [–0.130, 0.010] |
|  | Acceptance *×* Comprehensibility (*XW*_1_) | 0.018 [–0.019, 0.054] | –0.012 [–0.096, 0.072] | 0.024 [–0.050, 0.097] |
|  | Acceptance *×* Footing in the world (*XW*_2_) | –0.095 [–0.136, –0.055]*** | –0.042 [–0.124, 0.041] | –0.048 [–0.124, 0.028] |

* *p*<.050, ** *p*<.010, *** *p*<.001.

*Notes*. Bolded texts signal the presence of a between-group difference in the corresponding path coefficient. *β* is standardized path coefficient; *b* is unstandardized path coefficient. Analyses were based on the MLR estimator. Analyses were weighted. Model fit indices: RMSEA=0.048, 90% CI [0.043, 0.053], SRMR=0.033, CFI=0.977, TLI=0.961, χ²(32)=314.583. Acceptance, cognitive integration (comprehensibility, footing in the world), and symptoms were included as continuous variables and were standardized. Proximity to affected area was categorized as in non-affected area and in affected area (i.e., Adana, Adiyaman, Diyarbakir, Elaziğ, Gaziantep, Hatay, Kilis, Kahramanmaraş, Malatya, Osmaniye, and Şanliurfa). Demographic covariates included age, gender, and marital status.

**Supplementary Material 5**

*Associations Between Acceptance, Cognitive Integration, and Psychological Symptoms (Stratified by Property Damage Status)*

| **Dependent variables** | **Predictors** | **Property damage status** | | |
| --- | --- | --- | --- | --- |
|  |  | **No damage (*n*=7,214)**  ***β*_1_ [95% CI]** | **Damage (*n*=371)^1^**  ***β*_2_ [95% CI]** | **Difference test**  ***b*_1_ *– b*_2_ [95% CI]** |
| Anxiety symptoms (*Y*_1_) | Acceptance (*X*) | 0.072 [0.049, 0.095]*** | 0.077 [–0.023, 0.177] | –0.005 [–0.106, 0.095] |
|  | Comprehensibility (*W*_1_) | –0.206 [–0.232, –0.180]*** | –0.210 [–0.333, –0.086]** | 0.004 [–0.121, 0.130] |
|  | Footing in the world (*W*_2_) | –0.413 [–0.439, –0.387]*** | –0.403 [–0.516, –0.289]*** | –0.009 [–0.129, 0.110] |
|  | Acceptance *×* Comprehensibility (*XW*_1_) | 0.010 [–0.023, 0.044] | –0.033 [–0.189, 0.124] | 0.033 [–0.089, 0.155] |
|  | Acceptance *×* Footing in the world (*XW*_2_) | –0.082 [–0.116, –0.047]*** | 0.043 [–0.103, 0.189] | –0.103 [–0.218, 0.013] |
| Depressive symptoms (*Y*_2_) | Acceptance (*X*) | 0.096 [0.071, 0.120]*** | 0.054 [–0.065, 0.173] | 0.041 [–0.080, 0.162] |
|  | Comprehensibility (*W*_1_) | –0.151 [–0.180, –0.123]*** | –0.181 [–0.306, –0.055]** | 0.030 [–0.101, 0.160] |
|  | Footing in the world (*W*_2_) | –0.355 [–0.382, –0.328]*** | –0.373 [–0.483, –0.263]*** | 0.020 [–0.094, 0.134] |
|  | Acceptance *×* Comprehensibility (*XW*_1_) | 0.055 [0.020, 0.089]** | 0.022 [–0.149, 0.192] | 0.028 [–0.105, 0.162] |
|  | Acceptance *×* Footing in the world (*XW*_2_) | –0.102 [–0.138, –0.066]*** | –0.010 [–0.156, 0.136] | –0.079 [–0.195, 0.036] |
| PTSD symptoms (*Y*_3_) | Acceptance (*X*) | 0.094 [0.069, 0.118]*** | 0.099 [–0.012, 0.210] | –0.006 [–0.118, 0.106] |
|  | Comprehensibility (*W*_1_) | –0.195 [–0.223, –0.168]*** | –0.152 [–0.272, –0.032]* | –0.043 [–0.165, 0.079] |
|  | Footing in the world (*W*_2_) | –0.391 [–0.419, –0.364]*** | –0.366 [–0.480, –0.253]*** | –0.025 [–0.144, 0.094] |
|  | **Acceptance *×* Comprehensibility (*XW*_1_)** | **0.015 [–0.019, 0.050]** | **–0.173 [–0.328, –0.018]*** | **0.144 [0.024, 0.264]*** |
|  | **Acceptance *×* Footing in the world (*XW*_2_)** | **–0.091 [–0.128, –0.054]***** | **0.109 [–0.034, 0.252]** | **–0.161 [–0.274, –0.047]**** |

* *p*<.050, ** *p*<.010, *** *p*<.001.

*Notes*. ^1^ Results should be interpreted with caution, given the small sample size in the subgroup with property damage (4.89%). Bolded texts signal the presence of a between-group difference in the corresponding path coefficient. *β* is standardized path coefficient; *b* is unstandardized path coefficient. Analyses were based on the MLR estimator. Analyses were weighted. Model fit indices: RMSEA=0.048, 90% CI [0.043, 0.053], SRMR=0.033, CFI=0.978, TLI=0.962, χ²(32)=308.051. Acceptance, cognitive integration (comprehensibility, footing in the world), and symptoms were included as continuous variables and were standardized. Property damage status was categorized as no damage and damage. Demographic covariates included age, gender, and marital status.

**Supplementary Material 6**

*Associations Between Acceptance, Cognitive Integration, and Psychological Symptoms (Stratified by Interpersonal Loss Status)*

| **Dependent variables** | **Predictors** | **Interpersonal loss status** | | |
| --- | --- | --- | --- | --- |
|  |  | **No loss (*n*=5,991)**  ***β*_1_ [95% CI]** | **Loss (*n*=1,594)**  ***β*_2_ [95% CI]** | **Difference test**  ***b*_1_ *– b*_2_ [95% CI]** |
| Anxiety symptoms (*Y*_1_) | Acceptance (*X*) | 0.072 [0.046, 0.098]*** | 0.062 [0.020, 0.105]** | 0.010 [–0.040, 0.059] |
|  | Comprehensibility (*W*_1_) | –0.197 [–0.226, –0.168]*** | –0.233 [–0.286, –0.180]*** | 0.035 [–0.026, 0.096] |
|  | Footing in the world (*W*_2_) | –0.397 [–0.426, –0.368]*** | –0.437 [–0.488, –0.385]*** | 0.039 [–0.022, 0.100] |
|  | Acceptance *×* Comprehensibility (*XW*_1_) | 0.008 [–0.029, 0.045] | 0.014 [–0.054, 0.083] | –0.005 [–0.068, 0058] |
|  | Acceptance *×* Footing in the world (*XW*_2_) | –0.072 [–0.111, –0.033]*** | –0.089 [–0.159, –0.019]* | 0.010 [–0.055, 0.076] |
| Depressive symptoms (*Y*_2_) | Acceptance (*X*) | 0.092 [0.064, 0.121]*** | 0.084 [0.037, 0.130]*** | 0.009 [–0.046, 0.063] |
|  | Comprehensibility (*W*_1_) | –0.143 [–0.174, –0.111]*** | –0.178 [–0.233, –0.123]*** | 0.036 [–0.028, 0.099] |
|  | Footing in the world (*W*_2_) | –0.333 [–0.363, –0.303]*** | –0.391 [–0.446, –0.336]*** | 0.058 [–0.006, 0.123] |
|  | Acceptance *×* Comprehensibility (*XW*_1_) | 0.049 [0.010, 0.087]* | 0.070 [0.001, 0.139]* | –0.016 [–0.080, 0.048] |
|  | Acceptance *×* Footing in the world (*XW*_2_) | –0.096 [–0.136, –0.056]*** | –0.091 [–0.164, –0.019]* | –0.008 [–0.076, 0.060] |
| PTSD symptoms (*Y*_3_) | Acceptance (*X*) | 0.093 [0.065, 0.121]*** | 0.074 [0.032, 0.116]** | 0.019 [–0.031, 0.069] |
|  | Comprehensibility (*W*_1_) | –0.185 [–0.216, –0.153]*** | –0.211 [–0.265, –0.157]*** | 0.026 [–0.037, 0.089] |
|  | **Footing in the world (*W*_2_)** | **–0.362 [–0.393, –0.330]***** | **–0.440 [–0.492, –0.387]***** | **0.077 [0.015, 0.140]*** |
|  | Acceptance *×* Comprehensibility (*XW*_1_) | 0.017 [–0.022, 0.056] | –0.039 [–0.106, 0.027] | 0.046 [–0.017, 0.108] |
|  | Acceptance *×* Footing in the world (*XW*_2_) | –0.091 [–0.133, –0.048]*** | –0.036 [–0.101, 0.029] | –0.049 [–0.113, 0.015] |

* *p*<.050, ** *p*<.010, *** *p*<.001.

*Notes*. Bolded texts signal the presence of a between-group difference in the corresponding path coefficient. *β* is standardized path coefficient; *b* is unstandardized path coefficient. Analyses were based on the MLR estimator. Analyses were weighted. Model fit indices: RMSEA=0.047, 90% CI [0.042, 0.052], SRMR=0.033, CFI=0.978, TLI=0.964, χ²(32)=298.059. Acceptance, cognitive integration (comprehensibility, footing in the world), and symptoms were included as continuous variables and were standardized. Interpersonal loss status was categorized as no loss and loss (of family, relatives, friends, or someone the respondents know). Demographic covariates included age, gender, and marital status.
